# Use of the MATRICS consensus cognitive battery (MCCB) to evaluate cognitive deficits in bipolar disorder: A systematic review and meta-analysis

**DOI:** 10.1371/journal.pone.0176212

**Published:** 2017-04-24

**Authors:** Qijing Bo, Zhen Mao, Xianbin Li, Zhimin Wang, Chuanyue Wang, Xin Ma

**Affiliations:** 1 Beijing Key Laboratory of Mental Disorders, Department of Psychiatry, Beijing Anding Hospital, Capital Medical University, Beijing, China; 2 Center of Schizophrenia, Beijing Institute for Brain Disorders, Laboratory of Brain Disorders (Capital Medical University), Ministry of Science and Technology, Beijing, China; SPAIN

## Abstract

**Background:**

The Measurement and Treatment Research to Improve Cognition Schizophrenia Consensus Cognitive Battery (MCCB) has also been proposed for use in clinical trials to assess cognitive deficits in patients with bipolar disorder (BD). The aim of this study was to evaluate cognitive function assessed by the MCCB in BD.

**Methods:**

A literature search of the PubMed, Embase, PsycINFO, SCI, Cochrane Library databases and the Cochrane Controlled Trials Register was conducted. Case reports, reviews and meta-analyses were excluded and a systematic review of the remaining studies of cognitive function in BD was carried out. The cognitive outcome measure was the MCCB, including 7 domains and overall cognition. A random-effects model was applied.

**Results:**

Eighty eight studies were initially identified. Seven clinical studies comprising a total of 487 patients and 570 healthy controls (HC) were included in the meta-analysis. Patients with BD performed worse than HC in overall cognition and processing speed with a large effect size of >0.8; with a medium effect size (0.5–0.8) in attention, working memory, verbal learning and visual learning; and with a small effect size (0.2–0.5) in reasoning and problem solving and social cognition.

**Conclusion:**

Patients with BD performed worse than HC in overall cognition and all cognitive domains of the MCCB. Cognitive deficits in domains of processing speed and working memory are prominent in patients with BD. Our findings suggest that MCCB can be usefully applied in BD.

## Introduction

Cognitive impairment in schizophrenia has been noted for more than a century and has been a focus of research interest from the 1980s onwards [1]. Cognitive impairment is also recognized in major affective disorder: it has been recognized and studied in major depression for a long time [2], although studies of cognition in bipolar disorder (BD) are a more recent development [3]. Cognitive impairment is thus an important clinical symptom in bipolar disorder, and it may be that measures which produce improvements in cognition could provide better functioning and quality of life in patients [4]. Cognitive deficits in BD have been found in many domains of function, including attention, verbal learning/memory and executive function. Impairment of cognition has also been documented in first episode BD [5], in both bipolar type I (BD-I) and type II disorder (BD-II) patients, in the premorbid stage [6] and during periods of euthymic [7], with many of the findings being supported by meta-analysis [8–10].

Up to 167 instruments are currently available for the clinical assessment of cognitive dysfunction in schizophrenia, major depressive disorder and BD. However only two instruments frequently used in BD have been deemed appropriate [11]. In schizophrenia, a consensus clinical battery, The Measurement and Treatment Research to Improve Cognition in Schizophrenia (MATRICS) has been developed with the purpose of showing practical utility, good test–retest reliability and especially potential sensitivity to treatments designed to improve cognitive function in the disorder [12]. The MATRICS Consensus Cognitive Battery (MCCB) has been recommended by the United States Food and Drug Administration (FDA) to assess cognitive impairment as the primary outcome measure in registry trials of schizophrenia [13]. In contrast, no consensus has been reached on the optimal assessment of cognition in BD. Based on the clinical and cognitive overlap between schizophrenia and BD, some data have suggested that the MCCB might be a useful outcome measure for the assessment of cognitive function in BD [14]. The application of the MCCB in BD has been explored and proposed by the international society for bipolar disorder (ISBD) as a part of the battery for assessment of neurocognition in BD [15]. The objectives of this study were to assess cognitive function assessed by MCCB in BD.

## Materials and methods

### Search strategy

Studies evaluating cognitive performance in BD using the MCCB were identified by two authors by searching PubMed, Embase, PsycINFO, SCI, the Cochrane Library databases, the Cochrane Controlled Trials Register and the Chinese databases (CBM, CNKI, and WanFang). The search terms included: bipolar disorder, manic-depress, mania, psychosis, MCCB, MATRICS. All studies from inception to July 2016 were reviewed. Both indexing and free text search were used. No language restriction was set. The keywords were used in combination with the Boolean operators AND, OR, and NOT. The references of included articles were also screened to identify additional relevant studies.

Before initiating the study, our protocol for the review of cognitive function in bipolar disorder by MATRICS was registered online (http://www.crd.york.ac.uk/prospero/; registration number CRD42016046737 at the Preferred Reporting Items for Systematic Reviews and Meta-Analyses, PRISMA). PRISMA provides an evidence-based minimum set of items for reporting in systematic reviews and meta-analyses [16]. PRISMA Checklist has been included as supporting information “BPcognition-PRISMA checklist.doc” (S1 Checklist).

### Study selection criteria

Studies were included if performance was evaluated in BD using the MCCB. The specific meta-analysis criteria were as follows, i) Individuals were diagnosed with BD; if a study included patients with both schizophrenia and bipolar disorder, independent assessment of the bipolar disorder patients was required; ii) Cognitive performance was assessed using the MCCB; articles that used tests not covered by MCCB were excluded; iii) A healthy control (HC) group was included in the study. Review articles and case reports were not considered. If data were provided but were incomplete for the current aim, the authors were contacted by e-mail and asked to provide related information; where the authors did not respond the articles were excluded. Studies to be included for the meta-analysis were assessed by authors QJ-B and Z-M independently. Where the authors’ opinions differs a third author was consulted.

### Measures of cognition function

Cognitive function was measured according to the 7 cognitive domains of the MCCB derived from scores on 10 cognitive measures: speed of processing (Trail Making Test Part A; Brief Assessment of Cognition in Schizophrenia: Symbol coding; Category fluency test, animal naming), attention/vigilance (Continuous Performance Test: Identical Pairs), working memory (Wechsler Memory Scale, spatial span subset; Letter Number Span test), verbal learning (refers to immediate verbal memory, Hopkins Verbal Learning Test (HVLT)-Revised, immediate recall), visual learning (refers to immediate visual memory, Brief Visuospatial Memory Test-Revised), reasoning and problem solving (Neuropsychological Assessment Battery (NAB), mazes subtest), and social cognition (Mayer-Salovey-Caruso Emotional Intelligence Test (MSCEIT): managing emotions branch) [13, 17].

### Data analyses

Descriptive statistics for the sample characteristics and meta-analysis were performed with the software STATA (Stata Corporation, USA). A random-effects models was applied using the Metan command [18]. Cohen's method was performed to calculate standardized mean differences (SMD): effect sizes were calculated according to the difference between the means of BD and HC group divided by the pooled standard deviation (Cohen's d). Values over 0.8 are considered to represent a large effect size (ES), while values ranging from 0.5–0.8 and 0.2–0.5 represent medium and small ES respectively. The weighting to each SMD was calculated based on the sample size to compute the overall SMDs. Heterogeneity was assessed by the I^2^ test, which calculates the percentage of variation across studies due to heterogeneity rather than chance [19, 20]. Values of 25%, 50% and 75% correspond to low, moderate and high heterogeneity respectively. Tau^2^ was used as a measure of between study heterogeneity to undertake a random-effects. Publication bias was examined by the Egger's test [21] using the STATA Metabias command. Sensitivity analyses were performed by the metaninf command to investigate the influence of each individual study on the overall pooled ES estimate for each cognitive domain. To control for outlying outcomes from single study, the resiliency of every pooled ES to individual studies was estimated. The significance level was set at *P* < 0.05.

## Results

### Included studies

**Fig 1:**
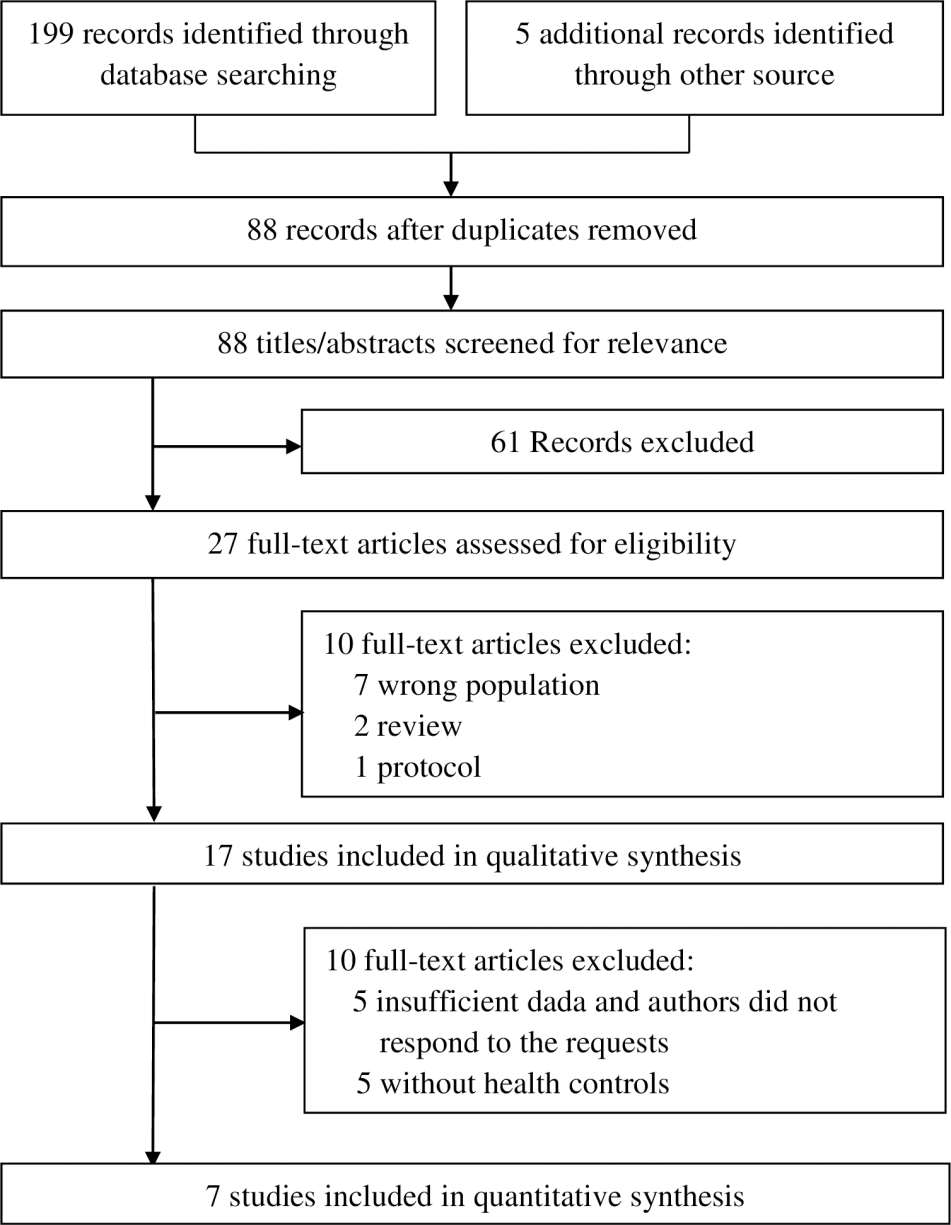
Literature search flow.

As shown in Fig 1, 88 studies were identified in the initial literature search. Of these, 17 were examined in detail, and that this led to 10 studies were excluded, leaving 7 in the meta-analysis (Table 1). Table 2 summarizes the objectives, samples, methods and results of the 10 studies that were excluded.

**Table 1:** Studies included in the meta-analysis.

Author (year)	Sample N	Male %	Age (year)	Education (year)	Medications	BD state	Clinical rating instruments
Lewandowski, 2016 [34]	BD-I 42	45	29.6±8.4	15.6±1.7	Detailed information about medication was not obtained, chlorpromazine (CPZ) equivalents were calculated	Did not classify, MADRS: 10.3±8.3 YMRS: 5.6±4.9	YMRS, MADRS, PANSS, MASQ, SHPS, MCAS
	HC 29	41	31.0±10.0	16.1±2.1			
Sperry, 2015 [35]	BD 56	42.86	30.5±8.16	14.9±2.0	Detailed information about medication was not obtained, chlorpromazine (CPZ) equivalents were calculated	Did not classify, MADRS: 12.13±7.23 YMRS: 5.21±4.67	YMRS, MARDS, PANSS, MCAS
	HC 57	40.35	25.67±6.42	15.1±2.1			
Burdick, 2014 [36]	BD-I 105 BD-II 31	50.0	40.8±10.6	14.1±2.0	45.7%Anticonvulsant, 74.1%Antipsychotic, 33.6%Antidepressant, 28.4%Lithium,	Did not classify, HAMD: 11.1±8.5 CARS-M: 5.5±7.0	HAMD, CARS-M, MSIF, SAS-Ⅱ
	HC 148	56.1	41.6±15.1	14.9±1.9			
Russo, 2014 [37]	BD-I 49 BD-II 9 BD NOS 6 6	50.0	41.2±10.5	Unknown	Unknown	64 euthymic: HRSD<15, CARS-M<8	HRSD, CARS-M TEMPS-A
	HC 109	53.2	37.9±11.6	Unknown	Unknown		
Van Rheenen, 2014 [38]	BD I 38 BD II 12	32.0	34.00±14.27	Unknown	Detailed information about medication was not obtained, dichotomous values indicated on or off antipsychotic agents/anticonvulsants, antidepressants, lithium or benzodiazepines were included in analyses	17 euthymic: YMRS< = 8 MADRS< = 8 33 symptomatic	YMRS, MADRS
	HC 52	38.5	38.44±13.02	Unknown			
Li, 2014 [39]	BD-I 59	44.1	31.2±8.9	14.4±2.8	39.0% atypical antipsychotics, 20.3%lithium, 37.3% sodium valproate, 10.2% other emotional stabilizer, 49.2% antidepressants, 8.5% benzodiazepine	29 euthymic, 30 symptomatic, According to DSM-IV	HAMD, GAF
	HC 27	44.4	29.5±4.1	13.7±2.6			
Burdick, 2011 [14]	BD-I 80	45	39.8±11.2	Unknown	50% anticonvulsant mood stabilizers; 80% antipsychotic medications; 33% lithium; 31% antidepressant	37 euthymic: HDRS< = 8 CARS-M< = 8 43 symptomatic	HRSD, CARS-M
	HC 148	56.1	41.6±15.1	Unknown			

PANSS: Positive and Negative Syndrome Scale, YMRS: Young Mania Rating Scale, MADRS: Montgomery-Asberg Depression Rating Scale, MASQ: Mood and Anxiety Symptom Questionnaire, SHPS: Snaith–Hamilton Pleasure Scale, MCAS: Multnomah Community Ability Scale, HAMD: Hamilton Depression Scale, CARS-M: Clinician-Administered Rating Scale for Mania, MSIF: Multidimensional Scale of Independent Functioning, SAS-II: Social Adjustment Scale-II, HRSD: Hamilton Rating Scale for Depression, TEMPS-A: Temperamental Evaluation of Memphis, Pisa, Paris, and San Diego-Autoquestionnaire

**Table 2:** Studies excluded in meta-analysis but included in qualitative synthesis.

**Garcia et al. 2016 [40]** No data presented on bipolar patients alone. **Objective:** To evaluate whether there are sex differences in relationship between childhood trauma and the clinical expression of the illness. **Sample:** 79 PD (schizophreniform disorder, schizophrenia, bipolar disorder, psychotic depression, unspecified psychotic disorder) and 59 HS. **Methods:** All participants were administered MCCB to assess cognition. Depressive, positive and negative psychotic symptoms, and global functioning were also assessed. History of childhood trauma was assessed using CTQ. **Results:** No sex differences were found in the CTQ scores. Childhood trauma was correlated to poorer social cognition. Emotional neglect and physical neglect had a more clearly association with more severe clinical symptoms and cognitive function.
**Nitzburg et al. 2016 [41]** Without health controls. **Objective:** To identify relations between coping strategies and real-world function in BD. **Sample:** 92 affectively-stable BD outpatients. **Methods:** Coping strategies were measured via the Brief COPE, real-world disability via the WHODAS-II, current symptoms, illness chronicity, and neurocognitive functioning via the MCCB. **Results:** Only verbal learning significantly predicted disability. Controlled for age, sex, illness chronicity, clinical symptoms, and neurocognitive function, behavioral disengagement and self-blame remained as predictors of disability,.
**Sanchez-Morla et al. 2016 [42]** No data presented on bipolar patients alone. **Objective:** To assess sensorimotor gating deficits in euthymic BD patients and analyze the relationships between PPI and clinical and cognitive measures. **Sample:** 64 patients with euthymic BD and 64 control subjects. **Methods:** Clinical characteristics and level of functioning were assessed in all participants using HDRS, YMRS and FAST. Cognition was evaluated using MCCB and the Stroop test as an additional measure of executive function. **Results:** Compared with controls, BD patients exhibited PPI deficits. Among BD patients, PPI was associated with the social cognition.
**Barbero et al. 2015 [43]** No data presented on bipolar patients alone. **Objective:** To explore whether HPT axis hormones or thyroid autoimmunity modulate cognitive function in individuals with early psychosis. **Sample:** 70 patients with a PD including 10 BD and 37 HS. **Methods:** Cognitive assessment was performed with MCCB. Plasma levels of TSH, FT4 and TPO-Abs and TG-Abs were tested. **Results:** In PD, higher FT4 levels were associated with better cognitive performance in attention/vigilance and overall cognition. Compared with non-affective psychosis, subjects with affective psychosis had increased FT4 levels and better cognitive performance.
**Kenney et al. 2015 [44]** No data presented on bipolar patients alone. **Objective:** To investigate the cognitive impairments during longitudinal course of four years after a FEP and the correlation of clinical performance and response to treatment. **Sample:** Twenty three individuals with psychotic illness including 2 BD and 21 healthy volunteers. **Methods:** Participants were assessed using the MCCB, PANSS and quality of life at illness onset and 4 years later. **Results:** Verbal learning and processing speed had shown poorer trajectory over the longitudinal course. Poorer clinical outcome was associated with greater deficits in reasoning and problem solving and social cognition at illness onset.
**Minor et al. 2015 [45]** No data presented on bipolar patients alone. **Objective:** To evaluate whether social and role functioning could be improved in six months and whether premorbid adjustment, baseline neurocognition and depression symptoms predicted functional improvement. **Sample:** All 78 participants were psychotic disorder patients in PREP^R^, including 7 BD. **Methods:** Premorbid adjustment was measured at baseline, while the Global Functioning Social and Role scales, MCCB, and Calgary Depression Scale were assessed at baseline and six months. **Results:** Improvements were evident in role functioning and social functioning. Premorbid adjustment and change in depression symptoms predicted role and social function change, except neuropsychological function.
**Russo et al. 2015 [46]** Without health controls. **Objective:** To examine the relationship between sleep dysfunction and neurocognition in BD. **Sample:** 117 BD patients. **Methods:** Neurocognitive function was assessed using MCCB. Sleep quality data were collected using ESS and PSQI. **Results:** Higher levels of sleep disruptions were correlated with a poorer performance in visual learning, working memory and social cognition, and negatively predicted working memory and social cognition.
**Cassidy et al. 2014 [47]** Without health controls. **Objective:** To explore the influence of the gene ANK3 on cognitive performance and brain structure among individuals with FEP. **Sample:** 173 patients with FEP, including 9 BD. **Methods:** Two single nucleotide polymorphisms (SNPs; rs1938526 and rs10994336) in *ANK3* in patients with FEP were genotyped. Cognition performance was assessed using MCCB. **Results:** Allele G of rs1938526 was associated with lower cognitive performance and significantly lower scores on verbal memory, working memory and attention. The significant effects of this SNP on cognition were vanished when adjusting for IQ.
**Kessler et al 2014 [48]** Without health controls. **Objective:** To compare the effects of right unilateral electroconvulsive therapy and algorithm-based pharmacologic treatment on neurocognitive performance in treatment-resistant BD depression. **Sample:** 73 treatment-resistant bipolar depression inpatients. **Methods:** General neurocognitive function was assessed with MCCB, and retrograde memory for AMI-SF before and shortly after a randomized 6-week trial. **Results:** Both groups exhibited improvements in all MCCB domains, with no significant differences between the groups. Improvements in neurocognitive profiles were significantly associated with reductions in depression ratings.
**Kessler et al. 2013 [49]** Without health controls. **Objective:** To assess the cognitive performance in treatment-resistant BD depression inpatients, to compare the cognitive function in BD-I and II patients, and to identify the factors influencing cognitive function. **Sample:** 19 BD-I and 32 BD-II inpatients with a major depressive episode. **Methods:** Participants were assessed with MCCB, the Wechsler Abbreviated Scale of Intelligence, the National Adult Reading Test, and MADRS, PANSS, GAF. **Results:** Neurocognitive impairments were evident in the BD-I and BD-II depression inpatients among all MCCB domains and more common in BD-I patients, manifested greater deficits in higher age patients.

PD: psychotic disorder, HS: healthy subjects, BD: bipolar disorder, PPI: prepulse inhibition, FEP: first-episode psychosis, PREP^R^: Boston’s Prevention and Recovery in Early Psychosis program, HPT: hypothalamic–pituitary–thyroid, MCCB: MATRICS Cognitive Consensus Cognitive Battery, CTQ: Childhood Trauma Questionnaire, DSM-IV: Diagnostic and Statistical Manual of Mental Disorders 4^th^ edition, WHODAS-II: World Health Organization Disability Assessment Schedule, 2nd Edition, FAST: Functioning Assessment Short Test, YMRS: Young Mania Rating Scale, HDRS: Hamilton Depression Rating Scale, ESS: Epworth Sleepiness Scale, PSQI: Pittsburgh Sleep Quality Index

Table 1 summarizes the characteristics of the 7 studies that met the eligibility criteria for meta-analysis in full. Six studies were published in English and one in Chinese. Five studies were conducted in the United States, one in Australia and one in China. Publication year ranged from 2011 to 2016.

A total of 487 BD patients and 570 HC were included in the meta-analyses. The sample sizes of the selected studies ranged from 71 to 284. The majority of patients were diagnosed with BD-I (76.6%), followed by BD-II (10.7%) and BD NOS (1.2%). In the remaining 11.5% BD subclassification was not reported. 30.2% of patients were diagnosed with euthymic BD and 21.8% with symptomatic BD, while diagnosis of the remaining 48% was not reported. The characteristics of the whole sample were described in Table 3.

**Table 3:** Sample characteristics of included 7 studies.

Characteristic	Patients		Controls	
	k	Mean (SD)	k	Mean (SD)
Age	7	36.71±16.03	7	38.59±16.66
Education (years)	4	14.53±2.20	4	14.96±2.11
Age of onset	4	21.53±9.20		N/A
Duration of illness (years)	2	11.46±8.21		N/A
HAMD	4	11.1±8.40		N/A
MADRS	3	11.51±8.53		N/A
YMRS	3	5.66±5.00		N/A
CARS-M	3	5.57±6.91		N/A
		% male		% male
Gender	7	45.36	7	51.04

k: number of studies, HAMD: Hamilton Depression Scale, MADRS: Montgomery-Asberg Depression Rating Scale, YMRS: Young Mania Rating Scale, CARS-M: Clinician-Administered Rating Scale for Mania.

Differences between BD and HC

**Fig 2:**
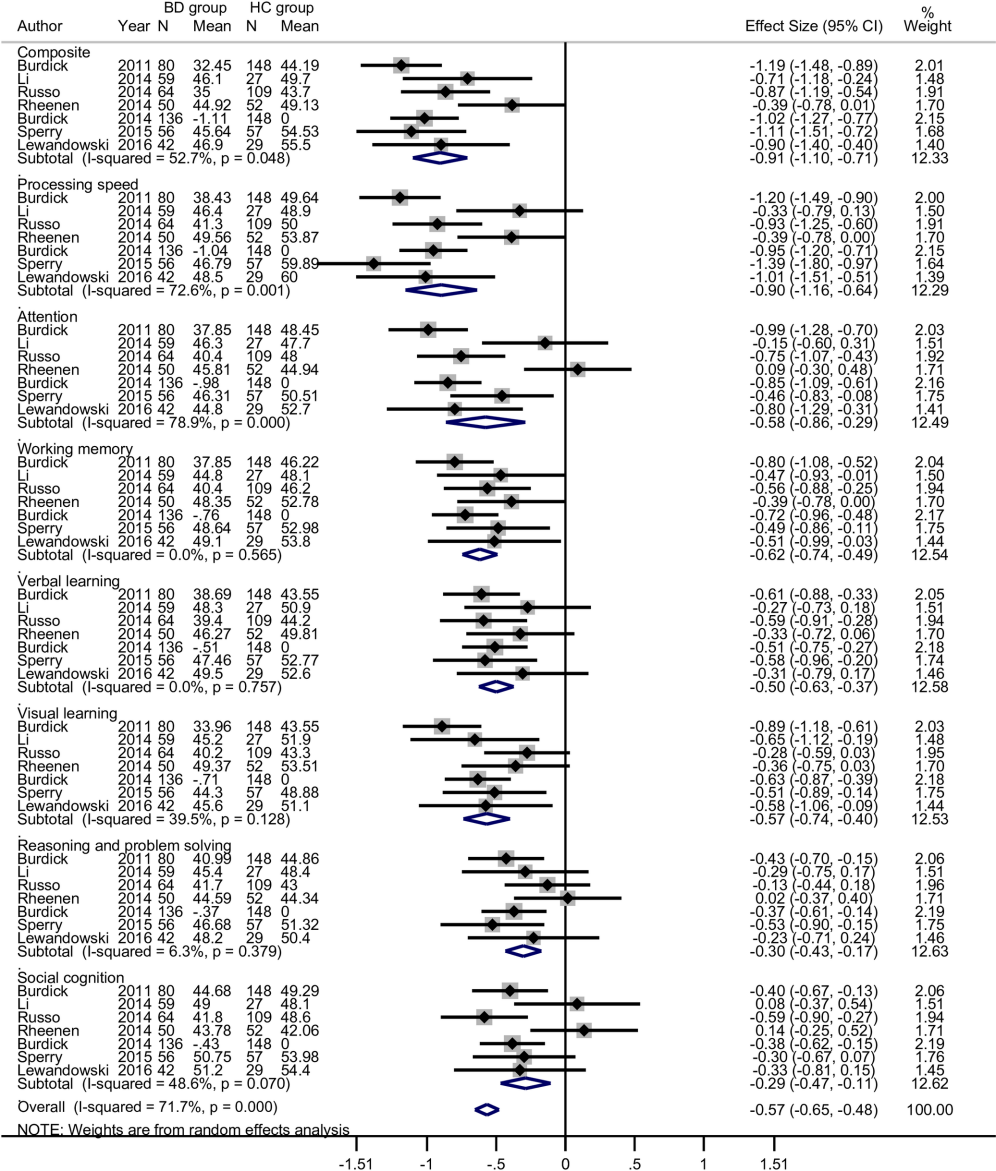
Forest plots of individual and pooled estimates of the SMD with random effect model between BD and HC groups for seven domains and composite scores of MCCB.

The results of the meta-analysis are summarized in Table 4; forest plots are shown in Fig 2. The BD patients performed more poorly than the HC in all 7 domains and in the composite score. Patients with BD exhibited worse performance than HC in overall cognition and processing speed with large ES (>0.8); in attention, working memory, verbal learning and visual learning with medium ES (0.5–0.8); in reasoning and problem solving and social cognition with small ES (0.2–0.5).

**Table 4:** Test for effect (Z, P), homogeneity (I^2^, Tau^2^), publication bias, and sensitivity analyses (pooled ES range; each study removed).

Cognitive domain	Z	P	I^2^	Tau^2^	Egger's test (t)	Sensitivity analyses
Processing speed	6.80	P<0.01	72.6%	0.09	2.28	0.35–0.45
Attention	3.94	P<0.01	78.9%	0.11	5.45	0.46–0.59
Working memory	9.59	P<0.01	0.00%	<0.01	2.85*	0.48–0.61
Verbal learning	7.82	P<0.01	0.00%	<0.01	1.99	0.54–0.69
Visual learning	6.63	P<0.01	39.5%	0.02	1.30	0.49–0.63
Reasoning and problem solving	4.60	P<0.01	6.30%	<0.01	1.32	0.65–0.83
Social cognition	3.12	P<0.01	48.6%	0.03	3.06	0.64–0.82
Composite	9.03	P<0.01	52.7%	0.04	2.73	0.34–0.45

### Heterogeneity

Composite scores were moderately heterogeneous across studies (P = 0.048, I^2^ = 52.7%, tau^2^ = 0.0358). In particular, attention had a high heterogeneity (P <0.001, I^2^ = 78.9%, tau^2^ = 0.1147) and processing speed had a moderate heterogeneity (P = 0.001, I^2^ = 72.6%, tau^2^ = 0.0856). Other cognitive domains displayed lower heterogeneity with I^2^s ranging from 0 to 48.6% (Table 4).

### Publication bias

Among the MCCB test scores, only working memory (t = 2.85, df = 6, P = 0.029) showed evidence of being influenced by publication bias (Egger's test, Table 4).

### Sensitivity analyses

Sensitivity analyses were performed for composite score and each significant cognitive domain. The statistical significance remained unchanged no matter which study was removed from the analysis. Examining the range of pooled ES in the composite score and each cognitive domain (Table 4) revealed that removal of each individual study from the pooled effect produced only a relatively little instability of the current findings.

## Discussion

This is the first meta-analysis examining the cognitive function of patients with BD as assessed using the MCCB. Among studies without language restrictions, it was possible to include seven studies comprising 487 patients with BD and 570 HC. Impairment in cognitive functions across all MCCB domains was detected in patients with BD compared to HC, with ES ranging from small to large. Cognitive deficits in domains of processing speed and working memory were found to be most prominent.

A previous meta-analyses found medium to large ES for differences between BD patients and HC in attention, processing speed, episodic memory and executive function [22]. Another meta-analysis reported impairment in verbal learning and memory and phonemic fluency with medium to large ES [23]. Findings have been similar in subsequent meta-analyses [9, 24, 25]. A systematic review of cognitive function in young BD patients during the first episode [6] found evidence of deficits across a range of domains, including verbal memory, attention, and executive functions, as did another systematic review of euthymic late-life BD subjects [26]. Our findings are consistent with these previous studies, but indicate impairment in a larger range of domains. Medium to large ES differences were observed in overall cognition, processing speed, attention, working memory, verbal learning and visual learning. Impairments in processing speed and working memory were found to be more prominent than those in attention, verbal learning and visual learning.

Social cognition is not usually considered in the assessment of cognitive function. However the MCCB includes social cognition measures, seemingly because of its negative effects on functional outcome in schizophrenia. The present meta-analysis finds that social cognitive deficits are also present in BD, in line with the results of existing studies [27, 28] and meta-analyses [29, 30]. While earlier work has found that social cognition impairment in BD has a medium ES, the current meta-analysis found it to be in the small range (-0.29), when assessed using the MCCB. Social cognition contains three main dimensions, namely emotion comprehension (ability to infer and appraise the emotional state displayed by other people), theory of mind (ability to ascribe mental states such as intents, desires, beliefs, knowledge and pretending) and attribution theory (interpretation of reward/punishment in relation to the individual’s thinking and behavior) [31, 32]. Further studies will be required to clarify the overall degree of social cognition impairment in BD and to further specify the degree of impairment in its different subdomains.

The MCCB was developed for schizophrenia and is frequently used in clinical trials in patients with this disorder. Nevertheless, the ISBD consider that it is also suitable for BD research and it has been included in the International Society for Bipolar Disorders–Battery for Assessment of Neurocognition (ISBD-BANC) [15]. The application of MCCB in BD patients began in 2011 and has increased gradually by year partly as a result of the ISBD recommendation. In comparison to previous meta-analyses of cognitive performance in BD patients, the present study focused on one instrument, avoiding differences in cognitive assessment instruments, helpful for pooling data for meta-analysis, and to be benefit of comparing with other psychoses such as schizophrenia.

Certain methodological limitations of the current study should be taken into account. Firstly, basing the analysis on group means in BD patients may tend to mask the effects of heterogeneity, ie the fact that some patients have no cognitive deficits while in others they are severe. Reporting of group means is the standard approach in neuropsychological studies of BD, and as a result there is no other way to pool data for meta-analytic purposes. Secondly, our meta-analysis does not provide any information regarding the longitudinal trajectory of cognitive dysfunction from the early stage to the chronic phase of disease. We note here that previous longitudinal meta-analysis of cognitive deficits in BD suggested that there was not enough evidence to indicate that cognitive dysfunction was a progressive course [33]. More in-depth studies will be required to explore the development of cognition and the magnitude of neuropsychological deficits in BD. Another limitation was that the potential effect of medications used in BD which impact cognition, e.g. mood stabilizers, antipsychotics, benzodiazepines, could not be examined. Similarly, the effects of BP subtypes (BP I vs BP II; psychotic vs nonpsychotic BD) and mood state (euthymic vs symptomatic) were not explored in this study due to the lack of data availability. The small number of studies included in the meta-analysis hindered analysis of moderator variables, which may influence cognitive performance. Lastly, various levels of heterogeneity were found among the cognitive domains and only the working memory domain displayed publication bias, which may indicate variability among the cognitive domains and the presence of bias or asymmetry in the literature.

## Supporting information

S1 Checklist “BPcognition-PRISMA checklist.doc”. (DOC)
